# Microsurgical resection of an enlarging lateral pontomedullary cavernous malformation

**DOI:** 10.3171/2019.7.FocusVid.1978

**Published:** 2019-07-01

**Authors:** Salomon Cohen-Cohen, Giuseppe Lanzino, Leonardo Rangel-Castilla

**Affiliations:** Department of Neurologic Surgery, Mayo Clinic, Rochester, Minnesota

**Keywords:** extended retrosigmoid, cavernous malformation, pontomedullary junction, video

## Abstract

The extended retrosigmoid approach provides an excellent corridor to the lateral aspect of the pontomedullary junction (PMJ).^1,2^ This video demonstrates a microsurgical resection of a progressive enlarging cavernous malformation (CM) of the PMJ. The patient is a 33-year-old woman with progressive symptoms, including right facial droop, left hemianesthesia, diplopia, and nystagmus. The patient underwent a right extended retrosigmoid approach with intraoperative neuronavigation and neuromonitoring. Lower cranial nerve dissection allowed access to the lateral PMJ. A longitudinal corticotomy was performed above the glossopharyngeal. The CM was removed in a piecemeal fashion. Postoperative MRI confirmed gross-total resection and the patient remained neurologically stable.

The video can be found here: https://youtu.be/K_TtiTo1RsQ.

## Transcript

In this video we present a microsurgical resection of an enlarging lateral pontomedullary cavernous malformation. The patient is a 32-year-old female presenting with 1-month history of intermittent left arm numbness and tingling. She did not have any significant past medical history and her examination was normal. On initial MRI we observed a small cavernous malformation on the right side of the pontomedullary junction. At that time, we decided to manage it conservatively and follow up with MRI. An MRI 1 year later demonstrated some enlargement of the cavernous malformation, and some of the symptoms worsened and some new symptoms appear like including facial numbness and mild imbalance. At that point the plan was still to monitor and manage it conservatively. However, symptoms significantly worsened 3 months after and now she presented with left upper and lower extremities weakness, right facial numbness and weakness, severe imbalance, diplopia, and nystagmus. Repeat MRI demonstrates further enlargement of the malformation, and it was decided to be time for microsurgical resection. The malformation goes to the surface of the brainstem laterally but also goes out to the fourth ventricle. We selected a lateral approach with an extended retrosigmoid craniotomy. Under general anesthesia we positioned the patient in a park bench position with the right side up using neuromonitor including the lower cranial nerves and intraoperative neuroimaging. We then preformed an extended retrosigmoid craniotomy. We opened the dura under the microscope and carefully reflected it anteriorly to protect the sigmoid sinus. We open the cisterna magna to drain CSF to help with brain relaxation and to minimize cerebellar trauma. With sharp dissection we dissected all the arachnoid planes and carefully mobilized the cerebellum away from the cranial nerves. We dissected around the VII–XII cranial nerves, carefully separating these nerves from the cerebellum. We were frequently checking our neuronavigation that we were on the right trajectory to the brainstem. Now we can observe hemosiderin on the lateral aspect of the pontomedullary junction and confirming the right trajectory to the cavernous malformation. We make a small incision longitudinal to the brainstem to preserve any long tracts. As soon as we puncture the brainstem we observe dark blood coming out under significant amount of pressure. Using micropickups and microdissectors we extend the incision into the cavity. We can observe that I have a lightened suction cannula on my left hand. By gently retracting the opening on the brainstem I start removing the cavernous malformation in a piecemeal fashion. Different from supratentorial cavernous malformation, malformations in the brainstem, there is minimal room for manipulation and it is very difficult to remove it circumferentially without injuring the brainstem. We continue the resection of the malformation with gentle traction of the brainstem. We can finally get to the posterior portion, which is always the most critical part, as we can leave some malformation behind. We inspect the cavity for any residual. As you can observe, the lighting suction is extremely useful to see the cavity. At this point, it is extremely difficult to inspect the cavity only with the microscope light. We can see that we are still leaving some malformation behind, and again doing a good inspection of the cavity we make sure we don’t leave any residual behind. As you can observe, I have a mouthpiece on the microscope and that allows me to always maintain an adequate focus while my two hands are helping to remove the malformation. This is the last portion of the cavernoma and I remove it with gentle traction and contratraction. I finally inspect the cavity seeing normal brainstem parenchyma. We do not remove the hemosiderin stain. Copious irrigation and the case is finish. Postoperative MRI demonstrates gross-total resection of the malformation. The patient remained neurologically as baseline and was discharged home 3 days after.

### Time points

2:25 Positioning

2:41 Dura opening

3:52 Corticotomy

4:35 Resection
